# The Fate of Tannins from Birdsfoot Trefoil and Their Effect on the Nitrogen Balance in Growing Lambs Fed Diets Varying in Protein Level

**DOI:** 10.3390/ani11010190

**Published:** 2021-01-14

**Authors:** Eleonora Seoni, Myriam Rothacher, Yves Arrigo, Silvia Ampuero Kragten, Giuseppe Bee, Frigga Dohme-Meier

**Affiliations:** 1 Agroscope, Route de la Tioleyre 4, 1725 Posieux, Switzerland; eleseoni@gmail.com (E.S.); myriam.rothacher@agroscope.admin.ch (M.R.); yvesarrigo@bluewin.ch (Y.A.); silvia.ampuero@agroscope.admin.ch (S.A.K.); giuseppe.bee@agroscope.admin.ch (G.B.); 2 Dipartimento di Scienze Zootecniche, Università di Sassari, Via E. De Nicola, 9, 07100 Sassari, Italy

**Keywords:** condensed tannin, forage legume, tannin balance, dietary protein level, digestibility, nitrogen excretion, body retention

## Abstract

**Simple Summary:**

Feeding forage legumes containing condensed tannins (CT) to ruminants has the potential to reduce urinary nitrogen (N) excretion, which is considered a significant contributor to environmental pollution. However, there are also indications that CT in the diet can reduce the feed intake. Furthermore, the fate of CT during digestion is still unclear. In the present study, the effect of CT from birdsfoot trefoil (BT) on the N excretion pathway and the CT excretion of lambs fed diets with different dietary crude protein (CP) levels were investigated. Regardless of the crude protein content of the diet, the results show that the CT-containing rations were eaten less frequently. Nitrogen excretion via urine decreased with the feeding of CT, but only in absolute terms and not in relation to the N intake. The CT balance indicates that not all CT that are ingested are also excreted. It seems that the CT change their structure when passing through the digestive tract.

**Abstract:**

Two experimental periods were employed to investigate the fate of ingested CT from BT and their effects on the intake, digestibility, and N balance in lambs fed diets differing in CP levels. In period 1, 24 lambs were fed a basal diet either treated with polyethylene glycol (PEG+) to deactivate CT or without polyethylene glycol (PEG−). In period 2, the same lambs were used in a 2 × 2 factorial design and fed either the basal diet (BP) or a high protein diet (HP), again treated with or without PEG. In both experimental periods, feeding lambs diets without PEG caused a reduction in the dry matter (DM) intake and an increase in the DM digestibility. Urinary and total N excretion decreased in lambs fed diets without PEG, but only in absolute terms and not in relation to the N intake. The total N intake, total N excretion, and body N retention were higher with a higher dietary CP level. Related to the CT intake, less soluble and protein-bound CT were excreted by lambs fed diets without PEG, and more protein-bound and fiber-bound CT were excreted by lambs fed BP diets. Regardless of the PEG treatment and CP level, not all CT that are ingested are also excreted. The effect of PEG treatment on the N excretion pathway was independent of the CP level.

## 1. Introduction

The effects of condensed tannins (CT) from legumes on ruminant nutrition, health, and production have been extensively studied and reviewed (e.g., [1,2]). Condensed tannins can have either beneficial or detrimental effects on ruminants, depending on the amount of CT that is consumed by animals, the type and chemical structure of the CT, and the composition of the diet [2]. Previous research has concentrated on the interactions between CT and dietary constituents, particularly proteins; however, little attention has been devoted to the fate of CT as they move along the digestive tract of ruminants. The results obtained from in vitro studies have demonstrated that the depolymerization of CT does not appear to take place under anaerobic conditions and may not occur in the rumen [3,4]. Furthermore, in vivo studies have demonstrated that CT were excreted in substantial amounts in the feces of sheep and goats [5], thereby suggesting that the digestion and absorption of CT do not occur in the gut. In contrast, studies with labeled CT have reported some modification or the disappearance of CT from the gastrointestinal tract in sheep and goats [6]. Kronberg et al. [7] even found very low concentrations of intact CT in the feces of sheep fed Chinese bushclover pellets. In addition, Quijada et al. [8], by analysing the concentration of CT in feed, digesta, and feces in lambs fed sainfoin pellets or hazelnut skins, reported a disappearance of up to 85%. However, so far, there has been no evidence of the degradation and absorption of CT in the digestive tract of ruminants and that quantitatively less intact CT are excreted than are ingested. The first objective of this study was to investigate the fate of CT from birdsfoot trefoil (BT; *Lotus corniculatus*) by monitoring possible changes in CT concentrations and soluble, protein-bound, and fiber-bound CT fractions after their passage through the digestive tract of lambs by conducting a CT balance. Thus far, the use of BT in ruminants has been investigated with respect to its effects on parasites [9], nitrogen (N) utilization [10], and meat quality [11]. The results of these studies have indicated that there can be significant variation in the effects of CT, not only due to the CT concentration, but also due to the composition of the basal diet, particularly the CP level, which has differed markedly among studies. Since CT displays a great affinity to protein, we hypothesize that the dietary CP level affects the impact of CT on the performance, digestibility, and N balance in lambs. In order to prove this hypothesis, we divided the study into two experimental periods. In period 1, we investigated the effect of CT as such. The reason we did this was based on the results of a previous experiment in which lambs that were fed different silages that were rich in CT, but differed in terms of the content of other nutrients, appeared to display a lower growth performance than lambs that were fed alfalfa [11]. The goal was to clarify whether the aforementioned effects only depended on CT or were due to the imbalance between crude fiber and low energy content that characterized the diets of lambs in the study of Girard et al. [11]. Therefore, in the present study, we equalized the dietary energy content and investigated, as a first step, the sole effect of CT from BT on the performance, digestibility, and N balance. In period 2, we increased the dietary CP to investigate the effect of a combination of CP and CT on the same parameters as those in period 1 and on the CT balance.

## 2. Materials and Methods

### 2.1. Animals, Diets, and Experimental Design

Two experimental periods were employed at Agroscope, Posieux, Switzerland, where male lambs of the white alpine sheep breed were fed diets comprising the CT-containing legume BT. Both experimental periods consisted of two series of 21 days each. In order to assess the effects of CT, the diets were tested with and without polyethylene glycol (PEG), which deactivates CT throughout the digestive tract. In period 1, 24 lambs (65 ± 12.8 days old and 21.7 ± 2.7 kg of BW) were allocated, by body weight (BW), to one of two groups of 12 animals each and fed a basal diet comprising BT silage, hay, and barley concentrate (15% CP on a DM basis; BP, Table 1). The diet was either treated with (PEG+) or without (PEG−) PEG. In period 2, the same 24 lambs (107 ± 12.8 days old and 27.2 ± 4.1 kg of BW) were subsequently divided by BW into four groups of six animals each and assigned to four dietary treatments. They were fed either the same basal diet as described in period 1 or a high protein diet (20% CP on a dry matter (DM) basis; HP). The HP diet was achieved by supplementing the BP diet with soybean meal, which caused a reduction of 13.9% in the CT content of the HP diets (Table 1). Then, the diets were supplemented with or without PEG, thereby yielding four different dietary treatments, consisting of BP-PEG+, HP-PEG+, BP-PEG−, and HP-PEG−. To prepare the PEG+ diets, PEG was dissolved in water (250 g PEG/L) and mixed with the silage to achieve a PEG:CT ratio of 1:1.

### 2.2. Metabolic Trial

To calculate the average BW during the metabolic trials, lambs were weighed at the beginning and end of both experimental periods. Between the two experimental periods, lambs were fed the basal diet. The diet in period 1 was calculated to obtain an average daily gain (ADG) of 200 g per day from 15–25 kg BW. The total energy content of the diet linearly increased from 4 to 6.8 MJ of net energy for meat production (NEv) [13]. Similarly, the diet in period 2 was calculated to obtain an ADG of 300 g per day from 25–35 kg BW. The total energy content of the diet linearly increased from 6.8 to 8.8 MJ of NEv [13].

For the study, 12 metabolic crates were available; thus, each experimental period, which consisted of two consecutive series, lasted for 21 days. During the first 14 days, the lambs were kept in pens (two animals per pen, kept separate during feeding) and were adapted to the experimental diets (adaptation period), as described previously [12]. Prior to the commencement of the collection period, the animals were allowed to become accustomed to the metabolic crates for two days. During the following seven days (the collection period), lambs were kept in metabolic crates that were fitted with a slatted floor and an inclined grid for the separate collection of urine and feces. During collection, the urine was divided into two parts. One part was acidified directly with 3 M sulfuric acid to avoid N losses. The feed intake and water consumption were recorded daily. Furthermore, individual diet components were sampled daily during the adaptation and collection periods, and feed refusal samples of each lamb were taken every day during the collection periods and subsequently pooled for each period to facilitate analysis of the chemical composition. Approximately 80 g of feces and 100 mL of acidified and un-acidified urine, respectively, were taken daily, pooled by lamb across the entire collection period, and stored continuously at −20 °C for subsequent analysis. Blood samples were collected from each lamb at the beginning and end of each collection period at 07:00, before feeding. Nine milliliters of blood per sample was taken from the vena jugularis, collected in VACUETTE^®^ tubes, and placed in a Z Serum Clot Activator (Greiner Bio-one GmbH, St. Gallen, Switzerland). One hour after the last blood collection, samples were centrifuged for 15 min at 3000 t/min and then for 2 min at 4000 t/min at room temperature. Thereafter, the serum was stored at −20 °C until urea analysis was conducted. All manipulations applied to the animals in the experiment were approved (No 2014_50_FR) by the Animal Care Committee of the Canton of Fribourg, Switzerland. The experiments were embedded in a larger study in which further traits related to meat quality and growth performance were investigated [12].

### 2.3. Laboratory Analysis

Prior to laboratory analysis, individual components of the diets were dried weekly at 60 °C for 24 h and ground to pass through a 1 mm screen (Brabender mill, No. 880804, Brabender, Duisburg, Germany). Refusal and feces samples of each animal were freeze-dried (Christ Delta 1-24 LCS, Osterode, Germany) and ground to pass through a 1 mm sieve (Brabender, Duisburg, Germany). The DM was quantified by drying at 105 °C for 3 h, and total ash was determined by dry ashing at 550 °C for 4 h [14]. Furthermore, the concentrations of total, soluble, protein-bound, and fiber-bound CT were determined in silage and feces samples using the HCl butanol method described by Terrill et al. [15], with the following modifications. The determinations were made in triplicate. A blank was conducted for each CT fraction of each sample by avoiding color development induced by heat (kept at 4 °C); thus, every absorbance was deduced by its corresponding blank. Cloudiness was often present in SDS blanks, but it disappeared after a few minutes at room temperature. Purified extracts of BT were used for calibration. Prior to CT analysis, feces samples were homogenized for 3 min with a 3D powder blender mixer (Turbula^®^ T2F, Bachofen AG, Wab group, Muttenz, Switzerland). The N content of feed and refusals was determined by the Dumas method [16], and CP was calculated as 6.25× N. The N content of feces and urine was determined by the Kjeldahl method [17]. Moreover, the urinary and plasma urea concentrations were analysed after enzymatic treatment was performed with urease and glutamate dehydrogenase (Urea kit UV 250, bioMérieux, Geneva, Switzerland) on an autoanalyzer (COBRAS Mira, Roche Diagnostic, Rotkreuz, Switzerland; standard: Calimat, bioMérieux), following the procedure set out by the manufacturer.

### 2.4. Statistical Analysis

The data collected in period 1 were subjected to one-way ANOVA using the SAS MIXED procedure (version 9.2). The model included the PEG treatment (PEG+ and PEG−) and the animal as the random variable. The data from period 2 were subjected to ANOVA using the SAS MIXED procedure (version 9.2). The model included the dietary CP level (basal and high), PEG treatment (PEG+ and PEG−), and one-way interaction as fixed factors and the animal as the random variable. The individual lamb was the experimental unit employed for the analysis of all data in both experimental periods. Additionally, least squares means were calculated and considered statistically significant at *p* ≤ 0.05, and tendencies were denoted at *p* ≤ 0.10. The full model of period 2 is expressed in the following equation:

Y_ijk_ = µ + p_i_ + t_j_ + pt_ij_ + S_k_ + ε_ijk_,

where µ is the general mean, p_i_ is the fixed effect of protein i, t_j_ is the fixed effect of CT j, pt_ij_ is the interaction of protein i with CT j, S_k_ is the random effect of animal k, and ε_ijk_ is the residual error.

## 3. Results

### 3.1. The Effect of PEG Treatment on the Intake, Digestibility, and N Balance in Lambs

The mean BW did not differ between treatments in period 1, but tended (*p* = 0.08) to be lower in lambs that were fed the PEG− diets compared to lambs that were fed the PEG+ diets in period 2 (Table 2).

Feeding lambs diets without PEG reduced (*p* < 0.05) the total DM and organic matter (OM) intake by 13.2% and 14.2%, respectively, in period 1 and by 7.6% and 7.5%, respectively, in period 2. Furthermore, the tap water intake tended (*p* = 0.09) to be lower in period 1 and was lower (*p* < 0.05) in period 2 in lambs that were fed the PEG− diet compared to lambs that were fed the PEG+ diets. The consumption of diets without PEG increased (*p* < 0.01) the apparent total tract digestibility of DM and OM by 7.0% and 6.2%, respectively, in period 1 and by 4.7% and 5.9%, respectively, in period 2. Lambs that were fed the PEG− diet ingested less (*p* < 0.05) N compared to lambs that were fed the PEG+ diet and this resulted in lower (*p* < 0.05) urinary N excretion in both experimental periods (Table 3).

**Figure 1:**
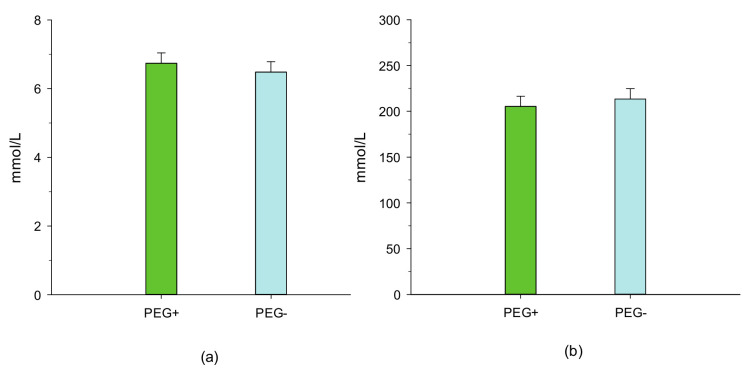
Effect of feeding birdsfoot trefoil diets treated with and without polyethylene glycol on plasma (**a**) (*p* = 0.55; SEM = 0.30) and urinary urea (**b**) (*p* = 0.61; SEM = 11.2) concentrations of lambs. PEG+, lambs that were fed a basal diet (15% crude protein on a dry matter basis) treated with polyethylene glycol, and PEG−, lambs that were fed a basal diet treated without polyethylene glycol.

**Figure 2:**
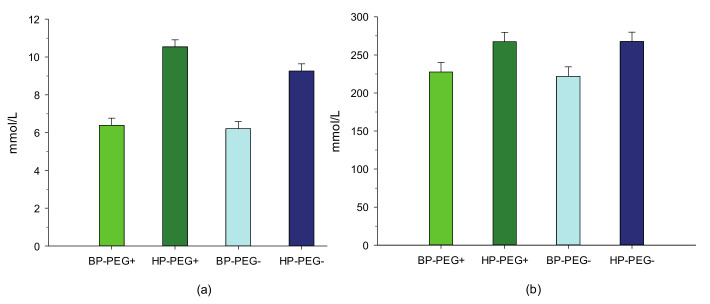
Effect of birdsfoot trefoil diets varying in crude protein level and treated with and without polyethylene glycol on plasma (**a**) (*p* = 0.16; SEM = 0.37) and urinary urea (**b**) (*p* = 0.82; SEM = 12.3) concentrations of lambs. BP-PEG+, lambs that were fed a basal diet (15% crude protein on a dry matter basis) treated with polyethylene glycol; HP-PEG+, lambs that were fed a high protein diet supplemented with soybean meal concentrate (20% crude protein on a dry matter basis) and treated with polyethylene glycol; BPPEG−, lambs that were fed a basal diet treated without polyethylene glycol; and HPPEG−, lambs that were fed a high protein diet supplemented with soybean meal concentrate and treated without polyethylene glycol.

As a result, the total N excretion tended (*p* = 0.10) to be lower in period 1 and was markedly lower (*p* < 0.05) in period 2. However, N retention in the body was not affected by PEG supplementation in the two experimental periods. When expressed as a percentage of N intake, feeding lambs diets without PEG had no effect (*p* > 0.05) on the total N balance in period 1, whereas the proportion of fecal N that was excreted was numerically (*p* = 0.13) greater in period 2. In both experimental periods, urea concentrations in plasma and urine were not affected by the treatment with PEG (Figure 1 and Figure 2).

### 3.2. The Effect of the Dietary CP Level on the Intake, Digestibility, and N Balance in Lambs

The total DM, OM, and water intake were 26.1%, 27.7%, and 44.6% greater (*p* < 0.001), respectively, in lambs that were fed the HP diet compared to lambs that were fed the BP diets (Table 2). The apparent total tract digestibility of DM and OM increased (*p* < 0.001) by 13.0% and 15.7%, respectively, in lambs that were fed the HP diet compared to lambs that were fed the BP diets. Although dietary CP supplementation affected the feed intake, the mean BW did not differ between treatments. Furthermore, the total N intake, fecal N excretion, urinary N excretion, total N excretion, and body N retention were greater (*p* < 0.01) when the HP diet was given (Table 3). However, when expressed as a percentage of N intake, fecal N excretion was lower (*p* < 0.001), while urinary N tended (*p* = 0.08) to be greater in lambs that were fed the HP diet compared to lambs that were fed the BP diet. Moreover, the urea concentrations in both plasma and urine were higher (*p* < 0.01) in lambs that were fed the HP diet.

Note that no interactions between the PEG treatment and CP level were determined for intake, digestibility, and N balance traits.

### 3.3. The Effect of the PEG Treatment and Dietary CP Level on the CT Balance

The soluble, protein-bound, fiber-bound, and total CT intakes were lower (*p* < 0.05) in lambs that were fed the PEG− diet compared to lambs that were fed the PEG+ diet and higher (*p* < 0.05) in lambs that were fed the HP diet than in lambs that were fed the BP diet (Table 4).

Furthermore, the level of soluble, protein-bound, and total CT excreted in feces was lower (*p* < 0.05) in lambs that were fed the PEG− diet than in lambs that were fed the PEG+ diet. The excretion of soluble CT tended to be greater (*p* = 0.07) in lambs that were fed the HP diet than in lambs that were fed the BP diet. Moreover, the fiber-bound CT excretion tended to be greater in the BP-PEG+ group than in the HP-PEG+ group, with intermediate values for the BP-PEG− and HP-PEG− groups (PEG treatment × dietary CP interaction, *p* = 0.05). When expressed as a percentage of the total CT intake, soluble and protein-bound CT excretion tended (*p* = 0.10) to be lower in lambs that were fed the PEG− diet compared with the PEG+ diet. The excretion of protein-bound and total CT tended (*p* = 0.10) to be lower in lambs that were fed the HP diets than in those that were fed the BP diets. Moreover, the fiber-bound CT tended to be greater in BP-PEG+ than in HP-PEG+, with intermediate values for BP-PEG− and HP-PEG− (PEG treatment × dietary CP, *p* = 0.05).

## 4. Discussion

### 4.1. The Effect of CT from BT on the Intake, Digestibility, and N Balance in Lambs

The results of our study confirm the commonly held view that feeding CT is associated with adverse effects on the feed intake [18], as the total DM and OM intakes were lower when the dietary CT were not deactivated by PEG. The negative effects of dietary CT on intake could be because of the reduced palatability of the diet. During the chewing process, CT react with salivary glycoproteins or directly with the taste receptors, causing an astringent sensation in the mouth. This sensation results in negative feedback, which induces the animal to reduce the consumption of feed that contains CT [19]. However, the literature provides contradictory results. Scharenberg et al. [20] found that, despite having elevated CT levels, sainfoin was more palatable than BT when fed to lambs, whereas studies with quebracho and chestnut tannin extract fed at 0.45% or 1% of DM, respectively, showed no effect on the DM intake in lactating cows [21]. Apart from the concentration, the biological effects of CT may differ, depending on the chemical structure of CT among plants and animal species and the diet composition [2]. Hence, the effect of CT on the feed intake depends on the balancing of these aspects. However, according to the literature, the negative effect of CT on the DM intake at the proportions used in this study was unexpected; currently, no evident reasons explaining these effects can be provided.

In addition to a reduction in feed intake, it is held that CT may also affect the digestibility of the diet, either by binding the digestive enzymes or by binding feed nutrients [18]. However, our findings disagree with this statement because the digestibility of DM and OM was found to be greater in lambs that were fed the PEG− diet compared with lambs that were fed the PEG+ diet in both experimental periods. This finding appears to be related to the lower DM and OM intake observed for these animals. Furthermore, improvements in digestibility occurred because the lower feed intake probably affected rumen turnover by reducing the rumen feed passage rate, which in turn increased the residence time in the digestive tract, thereby allowing more time for digestion [22].

Condensed tannins from legumes commonly reduce N excretion in urine as they cause a shift in excreted N from the urine to the feces [10,23]. The latter is of practical relevance for the environment, as urinary N is much more vulnerable to ammonia emission during manure storage [24]. A decrease in urinary N excretion is probably associated with the protein-binding properties of CT, which protect dietary protein from microbial degradation in the rumen and can ideally increase the proportion of amino acids (AA) available for post-ruminal absorption [1]. However, there is no clear evidence on whether the protein–CT complex formed in the rumen is dissociated, digested, or absorbed by the animal. Barry and McNabb [25] found that when sheep were fed with BT (22 g CT/kg DM) and *Lotus pedunculatus* (55 g CT/kg DM), the duodenal non-ammonia flow and absorption of AA were greater compared to those of sheep that were fed the same diet supplemented with PEG. Feeding CT from sainfoin (77 g CT/kg DM) decreased rumen protein degradation and increased the plasma level of essential AA [23]. In the present study, CT only had small effects on the N balance, both in absolute terms and when expressed as a percentage of the N intake. There was a weak trend for increased fecal N losses (relative to the N intake), but no effect on urinary excretion and N retention in the body in the BP-PEG− and HP-PEG− groups was observed. Moreover, the numerically lower plasma urea concentrations observed in the BP-PEG− and HP-PEG− groups than in the BP-PEG+ and HP-PEG+ groups are not consistent with a reduction in ruminal protein degradation, because the BP-PEG− and HP-PEG− groups had a lower intake of N compared with the BP-PEG+ and HP-PEG+ groups. Therefore, an improvement in the metabolic protein supply cannot be confirmed. A possible reason for this is that, in the present study, the dietary CT level might not have been sufficiently elevated to exert a marked effect on the N balance in lambs.

### 4.2. The Effect of the Dietary CP Level on the Intake, Digestibility, and N Balance in Lambs

In the present study, lambs that consumed HP diets had a greater DM and OM intake. The CP content of the diet is often positively related to the DM intake [26]. As the rate of breakdown and passage of the digesta increases, the feed intake increases accordingly. Furthermore, the daily water intake was also greater in lambs that were fed the HP diet compared to lambs that were fed the BP diet. This is probably because of the greater DM intake observed for these animals. There is normally a close relationship between the amount of water and the amount of feed consumed by herbivores in the fact that, with an increasing feed intake, water consumption also increases [27]. Hence, greater dietary CP stimulates not only the DM and OM intake, but also the water intake. The apparent total tract DM, OM, and N digestibility increased significantly in lambs that were fed the HP diets, probably due in part to the higher digestibility of soybean meal that was used at the expense of BT silage and hay in the HP diets. Our findings are in agreement with those of Dabiri and Thonney [28], who showed that the CP digestibility was higher for lambs that were fed a diet with 17% CP than for lambs that were fed diets containing 13% or 15% CP (DM basis). This could be attributed to an increase in the dietary CP concentration, which might produce an adequate N concentration for rumen microbes [29], thereby improving DM and OM digestibility.

In addition, the daily N intake was greater when HP diets were offered, which resulted in greater urinary and fecal N excretions, urinary urea N excretions, and N retention. Increased urinary N loss is the most commonly observed effect of a high CP intake. The greater loss of urinary N observed in lambs that were fed the HP diet is in agreement with the results of Cole [30]. An increased CP supply without a concomitant increase in energy supply leads to increased ruminal ammonia concentrations at a rate greater than that which ruminal microbes are able to metabolize, thereby leading to excess ammonia being absorbed by the ruminal wall. This results in increased urinary N and urinary urea N, as the excess ammonia is converted to urea and excreted in the urine. Moreover, N retention was closely related to the dietary CP intake, as it was greater in lambs that were fed the HP diet compared to lambs that were fed the BP diet. This finding can be attributed to the greater OM and N digestibility observed in lambs that were fed the HP diet, which improved the synthesis of microbial protein and resulted in a greater capture of ammonia that would have otherwise been lost as urea in the rumen, as suggested by Adesogan et al. [31]. When expressed as a percentage of N intake, lambs that were fed the HP diet excreted less fecal N, whereas urinary N excretion tended to be greater compared to lambs that were fed the BP diet. This reduction in fecal N excretion was again the result of improvements in dietary DM and N digestibility, which was in addition to the observed increase in body N retention. An increase in plasma urea concentrations with an increasing dietary CP level is a result that is in accordance with observations in other studies [28,30]. The plasma urea level represents an indicator of protein metabolism and is positively correlated with ingested CP [32]. The ammonia produced in the rumen due to the extensive degradation of dietary CP is absorbed by the rumen wall and then carried by the bloodstream to the liver, where it is converted to urea. Therefore, the increased blood urea concentrations observed in our study presumably reflect a lower N utilization efficiency for lambs as a consequence of an excessive supply of dietary CP, which leads to increased urea synthesis in the liver.

As previously mentioned, no significant interaction between CP and CT levels was determined for the intake, digestibility, and N balance of lambs. This implies that the dietary CP level did not affect the impact of CT on the aforementioned traits, probably because the dietary CT level was not sufficiently elevated to interact with the CP level of the diet.

### 4.3. The CT Balance

The fate of CT after ingestion is not fully understood in the existing literature. As previously mentioned, several studies have indicated that ruminal microorganisms cannot degrade CT, which allows CT to pass through the digestive tract without being absorbed in the intestine [3,4]. However, Perez-Maldonado and Norton [6] reported that CT could be absorbed or degraded along the digestive tract. In the present study, we monitored the fate of CT through the digestive tract by quantitatively collecting feces and determining the amount of excreted CT using the modified HCl butanol method based on Terrill et al. [15].

We found that most of the CT in the BT silage were bound to protein (54.8%), whereas the fiber-bound CT fraction represented the minor component (8.9%), with intermediate values for the soluble portion (35.8%). It is generally recognized that conservation methods, such as ensiling, reduce the extractability of CT and increase the insoluble portion of CT, with no effect on the total CT [33]. This is probably due to the partial disruption of plant cells as a result of physical chopping before ensiling, as well as due to the microbial fermentation that occurs during ensiling, which enables CT to react with other plant fractions and leads to an increase in the bound CT fraction [34]. The difference in the intake of soluble, protein-bound, fiber-bound, and total CT among treatments reflected the difference in the feed intake between the HP and BP groups and between the PEG− and PEG+ groups in the following decreasing order: HP-PEG+ > HP-PEG− > BP-PEG+ > BP-PEG−. Furthermore, differences in the CT content between HP (-13.9% of CT) and BP diets could be responsible for this difference.

Overall, we detected 35%–50% of the ingested CT in the feces of lambs. Of the three fractions, the soluble and protein-bound CT were affected more by PEG supplementation and displayed lower values in lambs that were fed the PEG− diet compared to lambs that were fed the PEG+ diet. The decrease in CT concentrations from feed to feces observed in this study is in line with the decrease of CT concentrations from feed to feces observed by Quijada et al. [8]. Perez-Maldonado and Norton [6] also found large decreases in CT in the fecal samples of sheep (86%) and goats (83%). Although the aforementioned studies did not conduct a balance study, the similarities with our findings could be explained by either the degradation or depolymerization of CT or both during passage through the small intestine. Furthermore, possible methodological issues, such as conformational changes of the ring structure, which would hinder the detection of CT by the HCl butanol method, have been given by Terrill et al. [35]. The exchange of CT among soluble, protein, and fiber-bound fractions after passage through the gastrointestinal tract could also be the reason for the numerically greater fiber-bound CT observed in the BP-PEG+ group than in the HP-PEG+ group.

## 5. Conclusions

The results of the present study suggested that the observed effects of dietary CT on the traits that were investigated were independent of the CP level and followed the same trend in both experimental periods. The lack of a substantial effect on traits relating to the N balance suggested that the content of CT in BT was probably too low. The investigation of the fate of CT after its passage through the digestive tract revealed that only 62% of the ingested CT could be recovered in the feces. These findings clearly indicate that CT changed their structure when passing through the rumen, stomach, and small intestine. This is in contrast to the widely proposed paradigm that CT pass through the digestive tract unchanged. Further research is required to define the optimum dietary CT concentrations, which could also enhance protein utilization in ruminants and, hence, reduce ammonia emissions.

## Figures and Tables

**Table 1:** Ingredients and gross chemical composition of the experimental diets used in experimental periods 1 and 2 ^1^.

	Experimental Diets	
Item ^2^	Basal Diet (BP)	High Protein (HP) Diet
Ingredient (% DM)		
Birdsfoot trefoil silage	53	45
Hay	41	36
Barley	6	6
Soybean meal		13
Chemical composition (g/kg DM) ^3^		
Dry matter	647 ± 61	686 ± 65
Organic matter	901 ± 15	913 ± 16
Crude protein	150 ± 54	202 ± 2
Crude fat	23 ± 8	22 ± 7
Neutral detergent fiber	429 ± 107	388 ± 97
Acid detergent fiber	291 ± 95	261 ± 85
Calculated energy and protein supply (per kg of DM) ^4^		
NEv (MJ)	5.6 ± 0.9	6.0 ± 0.9
APDE (g)	81 ± 3.6	96 ± 3.3
APDN (g)	107 ± 6.7	135 ± 6.2
Condensed tannins (g/kg DM)		
Soluble	3.3 ± 1.0	2.9 ± 0.9
Protein-bound	5.1 ± 1.3	4.4 ± 1.1
Fiber-bound	0.8 ± 0.3	0.7 ± 0.2
Total	9.3 ± 1.5	8.0 ± 1.3

^1^ Basal diet (BP) fed in experimental periods 1 and 2 treated with (PEG+) and without (PEG−) polyethylene glycol; high protein diet (HP), fed in period 2, treated with (PEG+) and without (PEG−) polyethylene glycol; means partially taken from Seoni et al. [12]; ^2^ DM, dry matter; ^3^ calculated from a chemical analysis of the individual diet components recorded daily during the adaptation and collection periods and pooled weekly; means ± standard deviation; ^4^ according to Agroscope [13]; NEv, net energy for meat production; APD, absorbable protein at the duodenum when the rumen fermentable energy (APDE) or nitrogen (APDN) is limiting microbial protein synthesis in the rumen.

**Table 2:** Effect of experimental diets on the intake, digestibility, and BW in lambs in experimental periods 1 and 2 ^1^.

	Period 1 ^2^				Period 2 ^3^							
	Treatment			*p*-Value ^4^	Treatment					*p*-Value ^4^		
Item	PEG+	PEG−	SE	PEG	BP-PEG+	HP-PEG+	BP-PEG−	HP-PEG−	SE	PEG	CP	PEG × CP
Body weight (kg) ^5^	24.8	23.9	1.24	0.44	33.2	35.4	30.8	32.7	1.59	0.08	0.14	0.90
Intake (g/kg of BW^0.75^ daily)												
Dry matter	67.6	58.7	3.02	0.05	85.5	108.0	79.2	99.7	3.92	0.02	<0.001	0.75
Organic matter	64.9	55.7	2.57	0.02	73.9	94.0	68.1	87.4	3.70	0.03	<0.001	0.91
Tap water intake (kg)	2.18	1.9	0.13	0.09	3.50	5.24	3.15	4.36	0.24	0.02	<0.001	0.29
Apparent digestibility (%)												
Dry matter	59.7	63.9	1.05	0.01	59.9	68.6	63.6	71.0	0.74	<0.001	<0.001	0.42
Organic matter	62.8	67.0	0.77	<0.001	58.0	68.1	62.3	71.1	1.05	<0.001	<0.001	0.54

^1^ BW^0.75^, metabolic body weight; PEG, polyethylene glycol; CP, crude protein; SE, standard error. ^2^ Period 1, lambs were 65 ± 12.8 days old; PEG+, basal diet treated with polyethylene glycol; PEG−, basal diet treated without polyethylene glycol. ^3^ Period 2, lambs were 107 ± 12.8 days old; BP-PEG+, basal diet treated with polyethylene glycol; HP-PEG+, high protein diet treated with polyethylene glycol; BP-PEG−, basal diet treated without polyethylene glycol; HP-PEG−, high protein diet and treated without polyethylene glycol. ^4^ Probability values for treatment with polyethylene glycol (PEG), the dietary crude protein level (CP), and the PEG × CP interaction. ^5^ Mean body weight measured before and after the collection period.

**Table 3:** Effect of experimental diets on the intake, digestibility, and BW in lambs in experimental periods 1 and 2 ^1^.

	Period 1 ^2^				Period 2 ^3^							
	Treatment			*p*-Value ^4^	Treatment					*p*-Value ^4^		
Item	PEG+	PEG−	SE	PEG	BP-PEG+	HP-PEG+	BP-PEG−	HP-PEG−	SE	PEG	CP	PEG × CP
N (g/kg of BW^0.75^ daily)												
Intake	1.88	1.60	0.07	0.01	2.24	3.75	2.09	3.30	0.14	0.01	<0.001	0.17
Feces	0.56	0.53	0.04	0.47	0.81	0.93	0.79	0.85	0.04	0.12	<0.01	0.29
Urine	0.72	0.62	0.04	0.05	0.78	1.46	0.70	1.22	0.06	0.02	<0.001	0.24
Total excretion	1.29	1.15	0.06	0.10	1.57	2.37	1.47	2.05	0.08	0.02	<0.001	0.19
Retention	0.60	0.45	0.06	0.12	0.63	1.34	0.57	1.20	0.09	0.19	<0.001	0.64
N (% of N intake)												
Feces	30.1	32.5	1.71	0.33	37.1	25.1	39.6	26.3	1.15	0.13	<0.001	0.58
Urine	38.8	39.4	2.33	0.85	35.7	39.2	34.2	37.5	2.24	0.40	0.08	0.96

^1^ N, nitrogen; BW^0.75^, metabolic body weight; PEG, polyethylene glycol; CP, crude protein; SE, standard error. ^2^ Period 1, lambs were 65 ± 12.8 days old; PEG+, basal diet treated with polyethylene glycol; PEG−, basal diet treated without polyethylene glycol. ^3^ Period 2, lambs were 107 ± 12.8 days old; BP-PEG+, basal diet treated with polyethylene glycol; HP-PEG+, high protein diet treated with polyethylene glycol; BP-PEG−, basal diet treated without polyethylene glycol; HP-PEG−, high protein diet and treated without polyethylene glycol. ^4^ Probability values for treatment with polyethylene glycol (PEG), the dietary crude protein level (CP), and the PEG × CP interaction.

**Table 4:** Effect of experimental diets on the fecal condensed tannin excretion of lambs over 7 days in experimental period 2 ^1^.

	Treatment				*p*-Value ^2^			
Item	BP-PEG+	HP-PEG+	BP-PEG−	HP-PEG−	SE	PEG	CP	PEG × CP
CT Intake (g/kg of BW^0.75^)								
Soluble	1.98	2.17	1.83	2.01	0.09	0.02	0.01	0.92
Protein-bound	3.03	3.33	2.80	3.07	0.13	0.02	0.01	0.91
Fiber-bound	0.50	0.54	0.46	0.50	0.02	0.02	0.01	0.91
Total	5.53	6.03	5.10	5.57	0.24	0.02	0.01	0.91
CT Feces (g/kg of BW^0.75^)								
Soluble	0.35	0.40	0.21	0.31	0.04	0.01	0.07	0.54
Protein-bound	1.60	1.40	1.17	0.99	0.17	0.03	0.30	0.97
Fiber-bound	0.78 ^x^	0.35 ^y^	0.46 ^xy^	0.50 ^xy^	0.11	0.45	0.10	0.05
Total	2.72	2.15	1.84	1.80	0.27	0.04	0.27	0.34
CT fecal excretion (% of total CT intake)								
Soluble	6.6	6.8	4.4	5.8	0.77	0.05	0.29	0.43
Protein-bound	27.6	21.2	21.2	16.5	4.09	0.10	0.10	0.80
Fiber-bound	14.4 ^x^	5.9 ^y^	9.3 ^xy^	9.1 ^xy^	1.93	0.64	0.04	0.05
Total	49.1	34.6	35.5	32.0	5.77	0.11	0.08	0.28

^1^ Period 2, lambs were 107 ± 12.8 days old; BP-PEG+, basal diet treated with polyethylene glycol; HP-PEG+, high protein diet treated with polyethylene glycol; BP-PEG−, basal diet treated without polyethylene glycol; HP-PEG−, high protein diet and treated without polyethylene glycol; CT, condensed tannins; BW^0.75^, metabolic body weight; PEG, polyethylene glycol; CP, crude protein; SE, standard error. ^2^ Probability values for treatment with polyethylene glycol (PEG), the dietary crude protein level (CP), and the PEG × CP interaction. ^x,y^ superscripts indicate a PEG × CP interaction; values within a row with different superscript letters tend to differ significantly at *p* ≤ 0.10.

## Data Availability

The data presented in this study are available on request from the corresponding author.
